# Artificial oxidative stress-tolerant *Corynebacterium glutamicum*

**DOI:** 10.1186/s13568-014-0015-1

**Published:** 2014-03-18

**Authors:** Joo-Young Lee, Hyo Jung Lee, Jiyoon Seo, Eung-Soo Kim, Heung-Shick Lee, Pil Kim

**Affiliations:** 1 Department of Biotechnology, The Catholic University of Korea, Bucheon 420-743, Gyeonggi, Korea; 2 Department of Biological Engineering, Inha University, Inchon 402-751, Korea; 3 Department of Biotechnology and Bioinformatics, Korea University, Jochiwon, Chungnam 339-700, Korea; 4 Present address: ST Pharm Co., Siheung 429-912, Gyeonggi, Korea

**Keywords:** Corynebacterium glutamicum, β-ketoadipate pathway, Oxidative stress-tolerance, pca gene clusters

## Abstract

We have reported a transcription profile of an adapted *Corynebacterium glutamicum* that showed enhanced oxidative stress resistance. To construct an artificial oxidative stress-resistant strain, gene clusters in the β-ketoadipate pathway, which were up-regulated in the adapted strain, were artificially expressed in the wild-type *C. glutamicum*. The wild-type strain was unable to grow under 2 mM H_2_O_2_ containing minimal medium, while the strains expressing *pca* gene clusters restored growth under the same medium, and the *pcaHGBC* expression showed the most significant effect among the gene clusters. The expressions of *pca* gene clusters also enabled the wild-type to increase its resistance against oxidative stressors, such as diamide and cumene hydroperoxide, as well as H_2_O_2_. The oxidative stress tolerance of the strain was correlated to the reactive oxygen species (ROS)-scavenging activity of the cell extract. The reason for the enhanced oxidative stress-resistance of *C. glutamicum* and its applications on the synthetic strain development are discussed.

## Introduction

*Corynebacterium glutamicum*, a Gram-positive bacterium with high GC-content that belongs to the order of *Actinomycetales*, is a well-known industrial strain for the production of various amino acids and nucleotides, such as lysine, glutamate, and inosine 5-monophosphate (IMP) (Eggeling and Bott [2005]). During the fermentation processes, the industrial strains encounter many artificially-driven stresses, such as temperature, pH, osmotic pressure, starvation, and oxidation. These kinds of stressors cause the loss of viability and cellular functions, which lower the productivity of bioprocesses (Li et al. [2009]). Because reactive oxygen species (ROS) such as superoxide radical, hydroxyl radical, and hydrogen peroxide are mainly formed during respiration, by the incomplete reduction of oxygen, and because oxidative stress by high oxidizing potential of ROS leads many damages, such as mutations, metabolic pathway disruption, and growth inhibition, oxidative stress is an unavoidable damage for oxygenic bioprocess of aerobic organisms (Fridovich [1998]).

**Figure 1:**
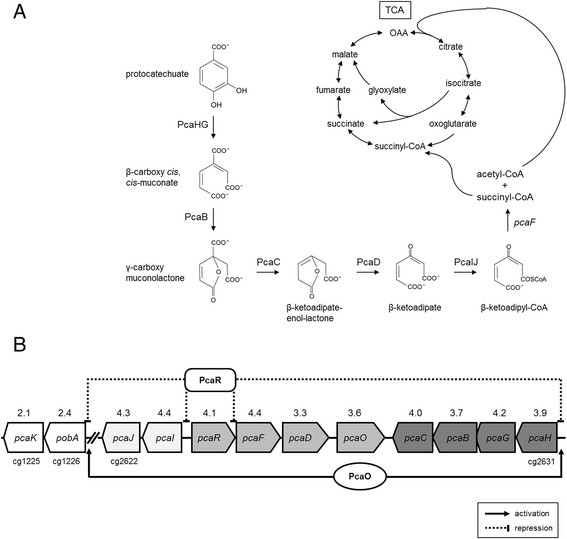
**Schematic diagram of the β-ketoadipate pathway (A) and distribution of*pca*gene clusters (B) in*Corynebacterium glutamicum*. (A)**. Descriptions and abbreviations: PcaHG, PCA 3, 4-dioxygenase; PcaB, β-carboxy-*cis*,*cis-*muconate cycloisomerase; PcaC, γ-carboxymuconolactone decarboxylase; PcaD, β-ketoadipate enol-lactone hydrolase; PcaIJ, β-ketoadipate:succinyl-coenzyme A transferase; PcaF, β-ketoadipate acetyl CoA acetyltransferase; TCA, tricarboxylic acid cycle. **(B)**. Genes organized as an operon are shown in the same color. Arrows indicate transcriptional activation and repression (Shen et al. [2012]; Zhao et al. [2010]). Numbers above genes indicate expression ratios of H_2_O_2_-adapted strain/wild-type from transcriptome results using RNA-seq (NCBI Gene Expression Omnibus access code: GSE41232) (Lee et al. [2013b]).

To understand the oxidative stress-induced responses of *C. glutamicum*, we have adaptively evolved the wild-type strain (ATCC 13032) under gradually increasing H_2_O_2_ conditions in a chemostat culture for 1,900 h, and have acquired a strain that was able to grow under 10 mM H_2_O_2_ conditions (Lee et al. [2013b]). The H_2_O_2_-adapted *C. glutamicum* strain (KCTC12280BP, i.e., HA strain) showed a distinguished transcriptome pattern (NCBI Gene Expression Omnibus access code: GSE41232). One of the unique transcriptome pattern of the adapted-HA strain was the up-regulations of genes involved in the degradation of aromatic compounds (*p*-coumarate, benzoate, quinate, shikimate, ferulate, vanillate, caffeate) in β-ketoadipate pathway, which could be linked to TCA cycle (Figure 1A), even though no aromatic compound was supplemented in the medium. This result brought about the theory that there might have been synthesis of aromatic antioxidants via the up-regulated β-ketoadipate pathway, and the ROS-scavenging activity of the intermediates in the β-ketoadipate pathway might have enabled the *C. glutamicum* HA strain to tolerate oxidative stress.

To verify this theory, we artificially expressed *pca* gene clusters in β-ketoadipate pathway (*pcaIJ*, *pcaFD*, and *pcaHGBC*) in the wild-type *C. glutamicum* and checked their survivability under oxidative stress conditions. The ROS-scavenging activities of the cell extracts were also estimated. Artificial oxidative stress-tolerant *C. glutamicum* is discussed based on the viewpoints of industrial applications.

## Materials and methods

### Strain and growth condition

*Corynebacterium glutamicum* ATCC 13032 containing vectors were grown in MCGC minimal medium composed of glucose 10 g, (NH_4_)_2_SO_4_ 4 g, KH_2_PO_4_ 3 g, Na_2_HPO_4_ 6 g, NaCl 1 g, sodium citrate dehydrate 1 g, biotin 200 μg, thiamine⋅HCl 1 mg, and minerals (FeSO_4_⋅7H_2_O 20 mg, MgSO_4_⋅7H_2_O 0.2 g, MnSO_4_⋅H_2_O 2 mg, FeCl_3_ 2 mg, ZnSO_4_⋅7H_2_O 0.5 μg, CuCl_2_⋅2H_2_O 0.2 μg, (NH_4_)_6_Mo_7_O_24_⋅4H_2_O 0.1 μg, Na_2_B_4_O_7_⋅10H_2_O 0.2 μg, and CaCl_2_ 70 μg) per liter (von der Osten et al. [1989]). Kanamycin (25 μg/mL) was supplemented to maintain vectors. Hydrogen peroxide (2 mM) was added to verify the growth against oxidative stress. Culture was performed at 30°C, 230 rpm in a 250 mL-Erlenmyer flask containing 50 mL medium. Cell growth was measured at O.D._600nm_ and was converted into biomass with an extinction coefficient of 0.250.

### Plasmid construction

Plasmid pSL360 (Park et al. [2004]), an empty expression vector carrying the P_180_ promoter, which induces constitutive overexpression of the cloned gene, was used to express *pca* gene clusters (Figure 1B). The *pca* gene clusters (*pcaIJ*: 5′-ga*ctgcag*t**gaa**cattacgttagcatgt-3′ and 5′-gg*ctgcag*ttaagcaactttgaaatc-3′, *Pst*I site italics; *pcaFD*: 5′-gg*atgcat*taaggatcaaaaaat**gaa**ccctc-3′ and 5′-gg*atgcat*ttaagcgaaatgctgtgc-3′, *Nsi*I site italics; *pcaHGBC*: 5′-aa*ctgcag*agacgca**gaa**aggtctc-3′and 5′-gg*ctgcag*ttactgaaggtctgacac-3′, *Nsi*I site italics). The native ribosome binding site (RBS) was modified with the consensus RBS sequence (bold) for high expression. The amplified DNAs of *pcaIJ* (1,404 bp), *pcaFD* (1,998 bp), and *pcaHGBC* (2,799 bp) were digested with *Pst*I or *Nsi*I, respectively, and were further ligated with *Pst*I-digested pSL360 (same overhang with *Nsi*I digestion) resulting in pSL360-*pcaIJ*, pSL360-*pcaFD*, and pSL360-*pcaHGBC*, respectively. The constructed vectors were electroporated (2 mm cuvette, 25 μF, 200 Ω, 2.5 kV) using an ECM 630 electroporation system (BTX, Holliston, MA, USA) into the wild-type *C. glutamicum,* after sequence verifications at a sequencing facility (Macrogen co., Seoul, Korea).

### Preparation of total RNA and RT-qPCR

Total RNA was extracted from *C. glutamicum* cells using TRIzol® reagent (Invitrogen, Carlsbad, CA, USA) and the NucleoSpin® RNA II Kit (Macherey-Nagel, Düren, Germany) according to the manufacturer’s instructions with the following modifications. *C. glutamicum* cells were harvested at an OD600 of 15, resuspended in TRIzol® reagent, and transferred to vials containing glass beads (acid-washed, 212–300 μm, Sigma-Aldrich, MO, USA). After cell disruption using Mini-Beadbeater-16 (Biospec, Bartlesville, PA, USA), the suspension was centrifuged, and the supernatant was applied to NucleoSpin® RNA II Kit (Macherey-Nagel, Düren, Germany). 50 ng of total RNA of *C. glutamicum* cells were used to cDNA synthesis using ReverTra Ace-α-® (TOYOBO, Osaka, Japan) according to the manufacturer’s instructions, respectively. THUNDERBIRD™ SYBR® qPCR Mix (TOYOBO, Osaka, Japan) and the Mx3005P QPCR System (Agilent Technologies, Santa Clara, CA, USA) were used for gene expression analysis. The RT-qPCR process was verified by melting curve and melting peak analyses. Relative quantity and standard error values from the expression analysis were calculated with MxPro-Mx3005P software ver. 4.10 (Agilent Technologies, Santa Clara, CA, USA). The following primers were used for detecting transcription level of *pca* genes: *pcaI*, 5´-acccagatgcagcaatga-3´ and 5´-gacgcggttgacgtaaattc-3´; *pcaJ*, 5´-atcggcatgcctacacttatc-3´ and 5´-gttcctcttcagttgggtaagg-3´; *pcaF*, 5´-ccactgggttccggtattt-3´ and 5´-gcgaaagcttcgttgagttc-3´; *pcaD*, 5´-aacttccgacaacaccttgg-3´ and 5´-cgatgacgcggaaatccttat-3´; *pcaH*, 5´-ggaccgttatgccaggtaat-3´ and 5´-ccgtaaactgacgaccatagag-3´; *pcaG*, 5´-cgctacgagcagtcgaatatc-3´ and 5´-aaaccgatgtggacgtaagg-3´; *pcaB*, 5´-ccgatctttatactccgaccttg-3´ and 5´-gcctccacgacaagaagatt-3´; *pcaC*, 5´-tcgctatgaaaccggaatgaa-3´ and 5´-cctgaaacttctcagtcacctc-3´; 16S rRNA, 5´-acccttgtcttatgttgccag-3´ and 5´-tgtaccgaccattgtagcatg-3´.

### Agar diffusion test

The tolerance of *C. glutamium* strains against various oxidative stressors were estimated by the agar diffusion test. Cells in log phase were mixed with 0.7% agar solution, and the mixture (3 mL) was poured onto 1.6% bottom agar plate containing 20 mL of BHI medium (Bacto™ Brain heart infusion 37 g/L, Cockeysville, MD, USA). A paper disc (6 mm diameter, Adventec, Tokyo, Japan) soaked with 20 μL of oxidative stressor (14% and 28% H_2_O_2_, 1 M diamide, or 10% cumene hydroperoxide, respectively) was placed on top of the agar, and the plate was incubated at 30°C for 24 h.

### Radical scavenging activity assay

Free radical scavenging activity of cell extract was estimated using 2, 2-diphenyl-1-picrylhydrazyl (DPPH), that is a stable free radical and decolorized when acquire an electron (Afify et al. [2012]). The bacterial cells grown to OD_600nm_ = 10 in BHI medium were harvested (5,000 rpm for 30 min at 4°C) and disrupted by Mini-BeadBeater16 (BioSpec, Bartlesville, OK, USA) to prepare the cell free extract. The supernatant was mixed with the same volume of ethyl acetate. After vigorous mixing, the ethyl acetate layer was separated by centrifugation and filtrated by 0.22 μm pore-membrane. The cell free extract was subjected to the free radical scavenging activity assay. Freshly prepared DPPH solution (2.8 mL) at a concentration of 5 mg/100 ml (in ethanol) were mixed with the cell free extract (200 μL) and incubated for 30 min in the dark at room temperature. Ethanol (200 μL) was the control. The absorbance for the sample (A_sample_) was monitored at 517 nm during incubation with 5 min intervals and further converted into the free radical scavenging activity according to the following equation:

(1) $$\mathrm{ROS}-\mathrm{scavenging}\;\mathrm{activity}\;\left(\%\right)=\left(A_{\mathrm{control}}-A_{\mathrm{sample}}\right)/A_{\mathrm{control}}\;\times \;100$$

The data were represented from the three biological repeated experiments.

## Results

### Effect of *pca* gene clusters expressions on acquired H_2_O_2_-tolerance

**Figure 2:**
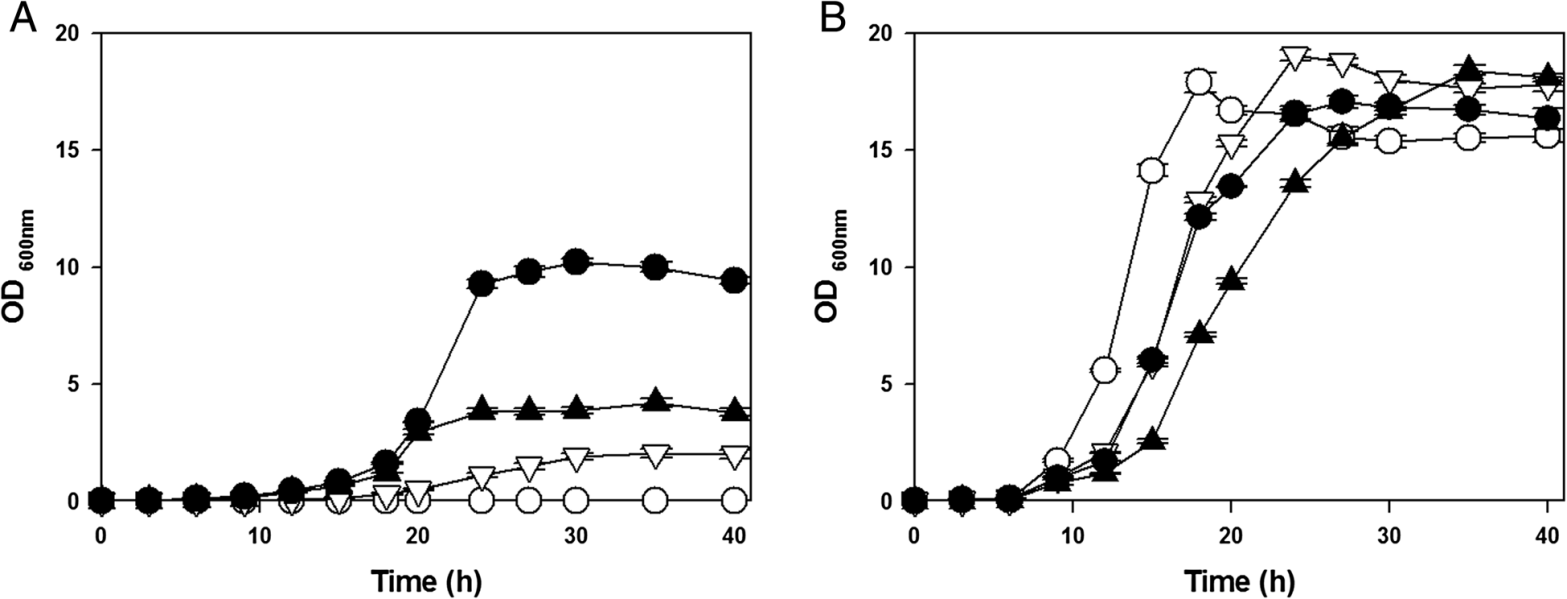
**Growth of*Corynebacterium glutamicum*strains in minimal medium (A) with and (B) without H**_**2**_**O**_**2**_**stress.** Symbols are indicated as following: wild-type strain carrying empty vector (−○–); wild-type expressing *pcaIJ* (−▽–); wild-type expressing *pcaFD* (−▲–); wild-type expressing *pcaHGBC* (−●–), respectively*.* Cells were cultured in MCGC minimal medium containing 25 μg/mL kanamycin, and the medium at the panel **(A)** additionally contained 2 mM H_2_O_2_.

To determine whether the expression of *pca* gene clusters affects the growth of *C. glutamicum* under the oxidative stress conditions, cells were cultured in the MCGC minimal medium with or without 2 mM H_2_O_2_. The control strain (wild-type *C. glutamicum* carrying empty pSL360 vector) was unable to grow when 2 mM H_2_O_2_ was present whereas the strain was able to grow till O.D. = 18 in 18 h without H_2_O_2_ (Figure 2). The wild-type strains carrying parts of *pca* gene clusters - *pcaIJ*, *pcaFD*, *pcaHGBC* – were able to grow under the oxidative stress conditions. The expression of *pcaHGBC* showed the most significant growth recovery (O.D. = 10.1 at 30 h), while the expression of *pcaIJ* showed the least (O.D. = 2 at 30 h). The expression of *pcaFD* showed an intermediate growth recovery (O.D. = 3.9 at 30 h).

For the verification of the expression of the *pca* gene clusters, the transcripts of the *pca* genes in the strains under no H_2_O_2_-stress conditions were analyzed by RT-qPCR (Table 1). All mRNAs of the *pca* genes those carried by the vector showed greater level than those in the wild-type. Only the fold of increase were varied depending on the clusters in the vector, that is, the mRNA levels of *pcaI* and *pcaJ* in the *C. glutamicum*(pSL360-*pcaIJ*) were 1.75- and 1.23-fold higher than those in the wild-type, mRNAs of *pcaF* and *pcaD* in the *C. glutamicum* (pSL360-*pcaFD*) were 40- and 42-fold higher, and mRNAs of *pcaH*, *pcaG*, *pcaB*, and *pcaC* in the *C. glutamicum* (pSL360-*pcaHGBC*) were 10.1-, 8.8-, 7.7-, 11.9-fold higher, respectively.

**Table 1:** **mRNA transcription levels of** ***pca*** **genes**

**Target gene**	**RNA-seq**^**a**^**(RPKM)**	**RT-qPCR**^**b**^**(relative fold)**			
	**WT**	**WT**	**+** ***pcaIJ***	**+** ***pcaFD***	**+** ***pcaHGBC***
*pcaI*	135.7	1.00 ± 0.39	1.75 ± 0.36	1.55 ± 0.50	0.61 ± 0.17
*pcaJ*	134.8	1.00 ± 0.15	1.23 ± 0.14	2.56 ± 0.27	1.10 ± 0.12
*pcaF*	220.9	1.00 ± 0.05	0.24 ± 0.03	40.2 ± 6.6	1.00 ± 0.15
*pcaD*	183.6	1.00 ± 0.18	0.25 ± 0.05	42.5 ± 5.9	1.00 ± 0.13
*pcaH*	172.0	1.00 ± 0.02	0.23 ± 0.01	2.61 ± 0.12	10.1 ± 0.95
*pcaG*	116.7	1.00 ± 0.01	0.23 ± 0.02	2.45 ± 0.16	8.83 ± 0.60
*pcaB*	217.5	1.00 ± 0.22	0.19 ± 0.04	2.31 ± 0.39	7.72 ± 0.42
*pcaC*	77.7	1.00 ± 0.02	0.21 ± 0.02	2.52 ± 0.23	11.9 ± 0.71

The cells were grown in MCGC minimal medium without H_2_O_2_-stress and harvested in the log phase for mRNA preparation.

^a^Adapted from (Lee et al. [2013b]) (NCBI Gene Expression Omnibus access code: GSE41232) RPKM (reads per kilo base per million).

^b^This study. Values are mean ± SD from three independent experiments.

### Effect of *pca* gene clusters expressions on the other oxidative stressors

**Figure 3:**
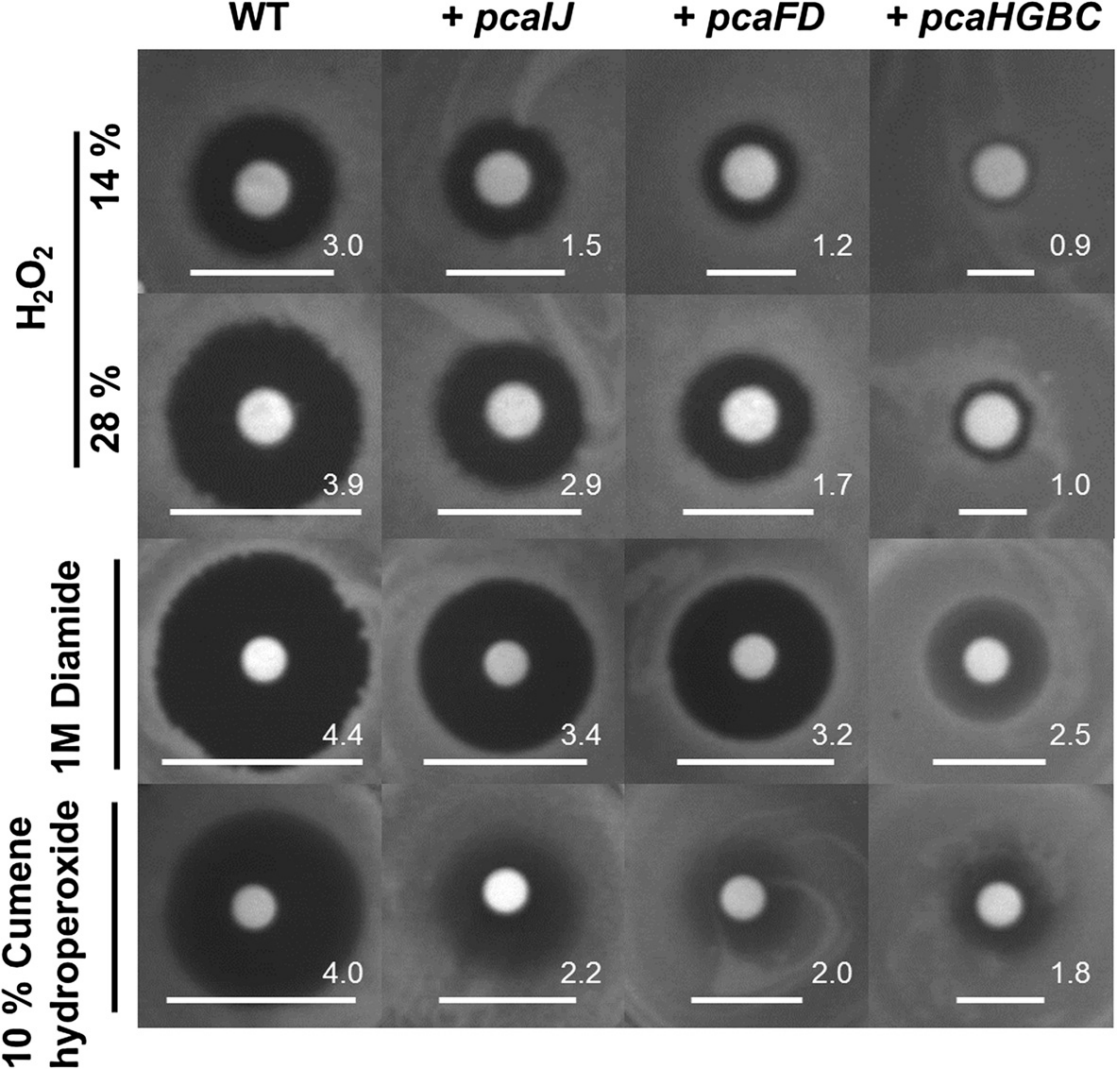
**Growth inhibitions of*Corynebacterium glutamicum*strains against oxidative stressors.** WT: *C. glutamicum* wild-type strains carrying empty vector, +*pcaIJ*: wild-type expressing *pcaIJ*; +*pcaFD*: wild-type expressing *pcaFD*; +*pcaHGBC*: wild-type expressing *pcaHGBC*. Vertical bars indicate the kind of oxidative stressors on the each paper disc. The agar plate contained BHI medium and a paper disc contained 20 μL of each stressor. The white bars and numbers indicate the size of each inhibition zone in centimeters.

To verify the effects of the expression of *pca* gene clusters on the tolerance against other oxidative stressors, agar diffusion tests were performed (Figure 3). The inhibition zones of the strain expressing *pca* gene clusters were smaller than that of the control against all tested oxidizing stressors (i.e., 14% and 28% H_2_O_2_, 1 M diamide, and 10% cumene hydroperoxide). The size of inhibition zones were in good agreement with the growth properties, that is, the smallest inhibition zone against the oxidative stress was found in the *pcaHGBC* expressing strain and the largest in the *pcaIJ* expressing strain, though still more tolerant than the control strain.

### Effect of *pca* gene clusters expressions on the intracellular ROS-scavenging activity

**Figure 4:**
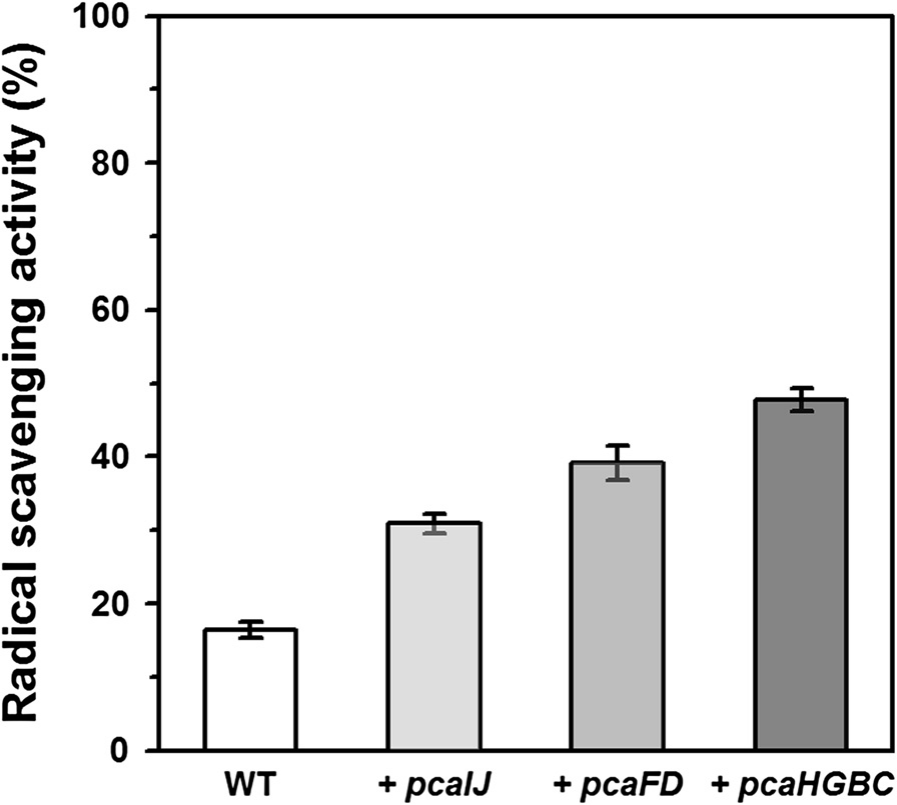
**ROS-scavenging activities of the cell free extracts from*Corynebacterium glutamicum*strains.** The cell extracts from the *C. glutamicum* wild-type strains carrying empty vector, *pcaIJ*-expressing vector, *pcaFD*-expressing vector, and *pcaHGBC*-expressing vector are indicated as WT, +*pcaIJ*, +*pcaFD*, and + *pcaHGBC*, respectively. Cells were harvested at OD = 10 in the BHI medium for cell extract preparations.

To understand the reason of the acquired oxidative stress-tolerance in the *pca* gene clusters expressing *C. glutamicum* strains, ROS-scavenging activity of the cell extract was estimated by DPPH assay (Figure 4 and Additional file 1: Figure S1). The cell extract from the *pcaHGBC* expressing strain showed 3-times greater ROS-scavenging activity (47.7 ± 1.6%) than that from the wild-type (16.4 ± 1.1%). The ROS-scavenging activities of the cell extracts from the *pcaFD* and *pcaIJ* expressing strains were 39.1 ± 2.3% and 30.9 ± 1.4%, respectively.

## Discussion

Constitutive overexpressions of *pca* gene clusters enabled wild-type *C. glutamicum* to tolerate oxidizing stressors, and *pcaHGBC* expression was the most effective among *pca* gene clusters (Figures 2 and 3). The ROS-scavenging activity of the cell extract was enhanced by the *pca* gene clusters expressions (Figure 4). Considering that the β-ketoadipate pathway by the *pca* gene clusters is involved in the degradations of aromatic intermediates (protocatechuate [PCA, 3,4-dihydroxybenzoate], vanillate [3-methoxy,4-hydroxybenzoate], and 4-hydroxybenzoate) and those were also found in natural herbs as antioxidants (Zheng and Wang [2001]), it would be reasonable to estimate that aromatic intermediates have been synthesized and contributed to ROS-scavenging activity to tolerate the oxidative stresses (Additional file 2: Figure S3).

The wild-type *C. glutamicum* transcribed the *pca* gene clusters at a low level, even though no aromatic carbon sources were present in the medium (NCBI Gene Expression Omnibus access code: GSE41232), and the transcription level of *pcaC* was the lowest among the *pca* genes: *pcaI*: 135.7; *pcaJ*: 134.8; *pcaF*: 220.9; *pcaD*: 183.6; *pcaG*: 116.7; *pcaH*:172.0; *pcaB*: 217.5; *pcaC*: 77.7 RPKM (reads per kilo base per million), respectively (Lee et al. [2013b]). This suggested *pcaC*, a putative 4-carboxymuconolactone decarboxylase, might have been the bottleneck step for synthesis of aromatic antioxidants in the wild-type strain, and overexpression of *pcaHGBC* might have been mainly responsible for the bottleneck of the pathway among all *pca* gene cluster expressions. Table 1 showed the mRNA levels of *pcaF*, *pcaD*, *pcaH*, *pcaG*, *pcaB*, and *pcaC* in the *C. glutamicum* (pSL360-*pcaIJ*) were even lower (0.19- ~ 0.25-fold) than those in the wild-type, and this might be the reason why the *pcaIJ* cluster expression showed the least effect of oxidative stress resistance among the tested *pca* gene clusters.

It is not clear the artificial *pca* gene clusters expressions have led the actual intracellular accumulation of aromatic intermediates. We were not able to detect the actual accumulation of recognizable aromatic metabolites from the methanolic extracts of the recombinant strains based on GC/MS analysis (Additional file 1: Figure S2), though few metabolites have been changed between the wild-type and the recombinant strains. The ROS-scavenging aromatic intermediates might not have been preserved enough to be detected. Okada and Okada reported that the supplementation of aromatic compounds in methanolic extract derived from broad bean increased the growth rate of human fibroblasts cells by ROS-scavenging activity (Okada and Okada [2007]), and the addition of 50 mg/L of a mixture of phenol carboxylic acids derived from wine (caffeate, ferulate, *p*-coumarate, gallate) was reported to stimulate bacterial growth (Rozes et al. [2003]). The addition of 0.1% gallate was also reported to enhance the aerobic growths of *Escherichia coli* ATCC 11775 and *Staphylococcus enteridis* ATCC 13076 1.5-fold and 2-fold, respectively (Lee et al. [2006]). These reports implied that an overexpression of the aromatic compound-synthetic pathway might be beneficial for the improved growth rates of industrial strains, considering the facts that cellular damages from ROS in aerobic bioprocess are unavoidable, and that aromatic compounds are able to scavenge growth-harmful ROS.

A number of microorganisms have been reported to produce aromatic compounds and their derivatives, via the aromatic compound-degrading β-ketoadipate pathway (Harwood and Parales [1996]). The biologically-beneficial properties of aromatic compounds as anti-oxidant, anti-cancer, and anti-inflammatory compounds have encouraged their synthesis using microorganisms. Advances in metabolic engineering and synthetic biology enabled the artificial biosynthesis of aromatic compounds (e.g. anthocyanins, caffeic acid, coumaric acid, hydroxybenzoic acid, ferulic acid, and genisteinin) using *E. coli* and *S. cerevisiae* (Yan et al. [2005]; Katsuyama et al. [2007]; Lin and Yan [2012]; Kang et al. [2012]). *C. glutamicum* has been reported to degrade aromatic compounds by β-ketoadipate pathway (Shen et al. [2004]; Shen and Liu [2005]; Merkens et al. [2005]; Brinkrolf et al. [2006]), and their regulations have been studied (Qi et al. [2007]; Haußmann et al. [2009]; Haußmann and Poetsch [2012]). Despite the industrial importance of *C. glutamicum*, there has been no report of the production of aromatic compounds from *C. glutamicum*. The findings in this study suggest that *C. glutamicum* is a potentially suitable host for the production of aromatic antioxidants via the β-ketoadipate pathway, as well as being suitable for further applications as an oxidative stress-tolerant host. Introduction of β-ketoadipate pathway of *C. glutamicum* into other species might be another application. The authors recently found the engineered *Escherichia coli* harboring greater intracellular ATP, even though useful for application (Kim et al. [2012]; Kim et al. [2011]), showed a growth defect (Lee et al. [2013a]) and intracellular accumulation of ROS was suspected as the reason of growth inhibition. The ROS scavenging activities from the *pca* gene clusters might enabled the engineered *E. coli* to reduce the ROS from the high ATP and to lead growth recovery.

In conclusion, the wild-type *C. glutamicum* acquired oxidative stress-tolerance based on the increased ROS-scavenging activity by introducing the β-ketoadipate pathway gene clusters, which suggests that the intermediates of the β-ketoadipate pathway contributed to the acquired tolerance. This finding could be further applied to develop a synthetic cell which is oxidative stress-tolerant and rapid growing industrial strain under oxidative stress conditions.

## Competing interest

The authors declare that they have no competing interests.

## Additional files

## Supplementary Material

Additional file 1: Figure S1. Kinetics of DPPH radical scavenging activity of cell free extracts of *Corynebacterium glutamicum*. **Figure S2.** The GC chromatogram of the methanolic extracts of *C. glutamicum* strains. Click here for file

Additional file 2: Figure S3. Summary for the expression effect of *pca* gene clusters on the artificial oxidative stress-tolerance in *Corynebacterium glutamicum*. Click here for file
