# Supplementary material for: Artificial oxidative stress-tolerant Corynebacterium glutamicum
Source: AMB Express. 2014 Mar 18;4:15. doi: 10.1186/s13568-014-0015-1 (PMC4052852; doi:10.1186/s13568-014-0015-1)
Supplement: Additional file 1: Figure S1. — Kinetics of DPPH radical scavenging activity of cell free extracts of Corynebacterium glutamicum. Figure S2. The GC chromatogram of the methanolic extracts of C. glutamicum strains. [file s13568-014-0015-1-S1.docx]

**Supplementary material:**

**Artificial oxidative stress-tolerant *Corynebacterium glutamicum***

Joo-Young Lee^1^, Hyo Jung Lee^1^, Jiyoon Seo^1^, Eung-Soo Kim^2^, Heung-Shick Lee^3^, and Pil Kim^1*^

^1^Department of Biotechnology, The Catholic University of Korea, Bucheon, Gyeonggi 420-743, Korea

^2^Department of Biological Engineering, Inha University, Inchon 402-751, Korea

^3^Department of Biotechnology and Bioinformatics, Korea University, Jochiwon, Chungnam 339-700, Korea

Running title: Artificial oxidative stress-tolerant *C. glutamicum*

Present address: J. Seo, ST Pharm Co., Siheung, Gyeonggi 429-912, Korea.

**Corresponding Author**

Pil Kim, Ph.D., Asso. Prof.

Dept. of Biotechnology, the Catholic Univ. of Korea,

Bucheon, Gyeonggi, Korea

T. +82-2-2164-4922; F. +82-2-2164-4865

Email: [kimp@catholic.ac.kr](mailto:kimp@catholic.ac.kr)

**Kinetics of DPPH radical scavenging activity of *C. glutamicum* strains**

**
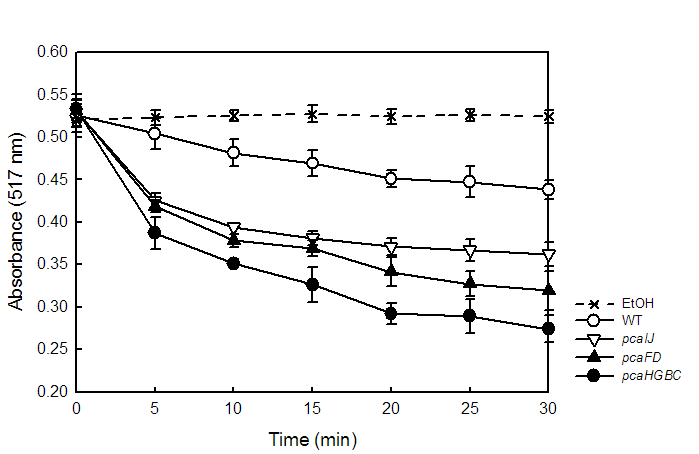
**

**Figure S1. Kinetics of DPPH radical scavenging activity of cell free extracts of *Corynebacterium glutamicum.***

Assay was performed as described in Materials and methods.

**The GC/MS chromatogram of the methanolic extracts of *C. glutamicum* strains**

**Method**

**Sample preparation.** To compare the intracellular metabolite profile, actively growing cells in MCGC minimal medium were harvested by centrifugation (4 °C, 3,600 rpm). Cells were disrupted using a Retsch MM400 Mixer mill (Retsch GmbH & Co, Haan, Germany) at 30 HZ s^-1^ for 10 min. The disrupted cells were then extracted with 1 mL methanol and were centrifuged at 4 °C and 12000 rpm for 5 min. The supernatant (100 *μ*L) was completely dried using a speed vacuum concentrator (Biotron, Seoul, Korea). The remained solute was oximated with 50 µL methoxyamine hydrochloride (20 mg mL^-1^ in pyridine) at 30 °C for 90 min, and silylated with 50 µL MSTFA at 37 °C for 30 min^^[[1]](#footnote-1)^^. 1 µL of sample was injected into the GC-TOF-MS (Leco, St. Joseph, MI, USA) analysis^^[[2]](#footnote-2)^^. Each peak was assigned based on the database (NIST05 MS Library, Wiley mass spectral database).

**GC-TOF-MS Analysis.** An Agilent 7890A GC system (Palo Alto, CA) equipped with an Agilent 7693 autosampler was connected to a time-of-flight Pegasus III mass spectrometer (Leco, St. Joseph, MI, USA), operating in electron ionization (EI) mode (70 eV). A HP-5MS column (30 m length×0.25 mm i.d. × 0.25 m film thickness, J & W Scientific, Folsom, CA, USA) was used with helium as a carrier gas at a constant flow of 1.5 mL/min. 1µL of the derivatized sample was injected with a splitless mode. The oven temperature was maintained at 75ºC for 2 min, increased to 300 º C at 15 º C/min, and then held at 300 º C for 3 min. The acquisition rate was set to 20 scans s−1 with a mass scan range of m/z = 50–1000. The injector and ion source temperatures were 250 º C and 230 º C, respectively

**Result**

**Figure S2. The GC chromatogram of the methanolic extracts of *C. glutamicum* strains.**

Each peak was verified by MS analysis (data not shown).

1. Li et al., “Study on the determination of polyphenols in tobacco by HPLC coupled with ESI-MS after solid-phase extraction”, J Chromatogr Sci 41: 36-40 (2003) [↑](#footnote-ref-1)
2. Siebenhandl et al., “Phytochemical profile of main antioxidants in different fractions of purple and blue wheat, and black barley”, J Agric Food Chem 55:8541-8547 (2007) [↑](#footnote-ref-2)
