# Supplementary figures and images for: Artificial oxidative stress-tolerant Corynebacterium glutamicum
Source: AMB Express. 2014 Mar 18;4:15. doi: 10.1186/s13568-014-0015-1 (PMC4052852; doi:10.1186/s13568-014-0015-1)

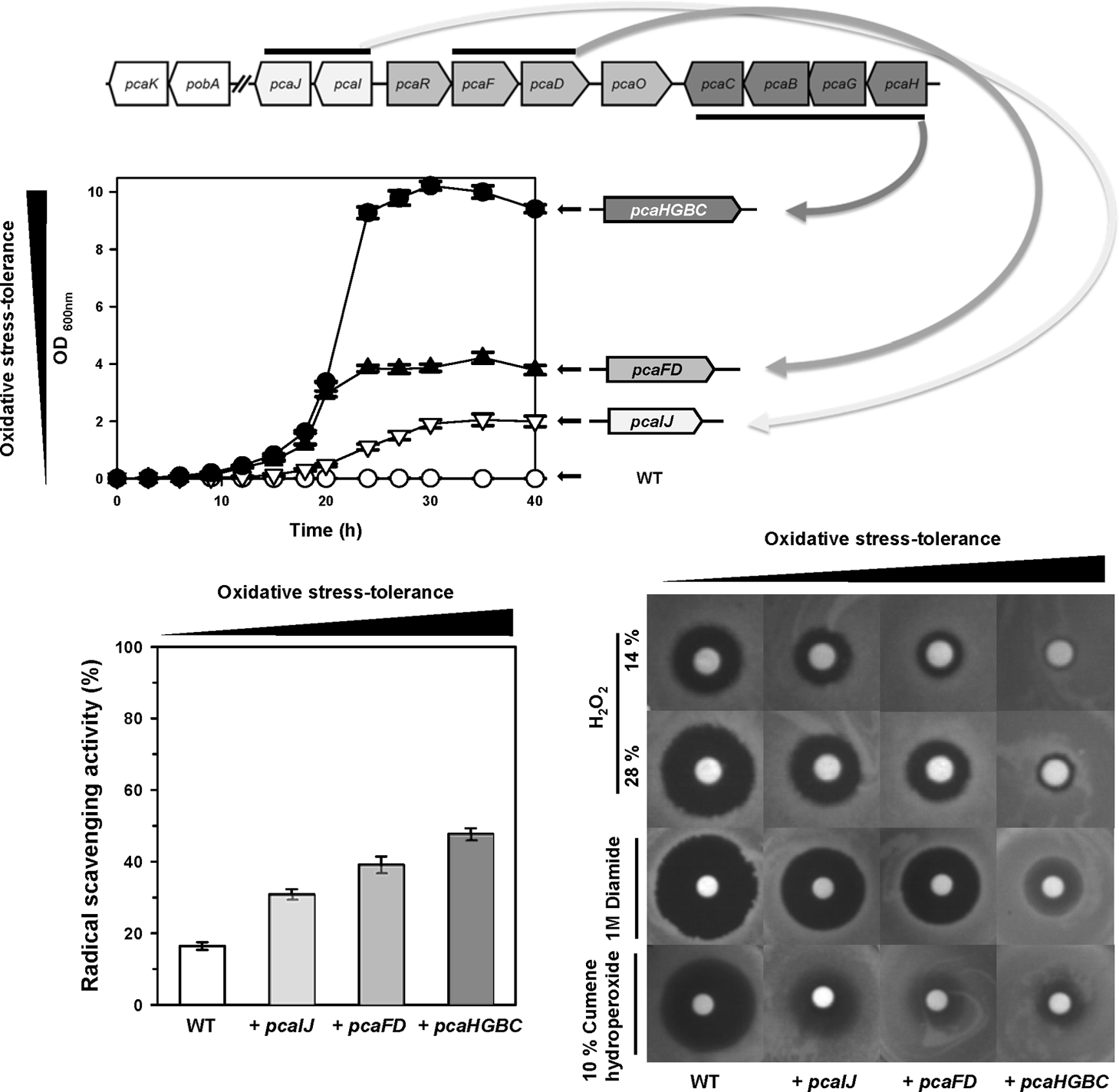

Supplement: Additional file 2: Figure S3. — Summary for the expression effect of pca gene clusters on the artificial oxidative stress-tolerance in Corynebacterium glutamicum. [file s13568-014-0015-1-S2.tiff]
